# Quantifying Variation in Soybean Due to Flood Using a Low-Cost 3D Imaging System

**DOI:** 10.3390/s19122682

**Published:** 2019-06-13

**Authors:** Wenyi Cao, Jing Zhou, Yanping Yuan, Heng Ye, Henry T. Nguyen, Jimin Chen, Jianfeng Zhou

**Affiliations:** 1 Institute Laser Engineering, Beijing University of Technology, Beijing 100124, China; wycao2020@163.com (W.C.); ypyuan@bjut.edu.cn (Y.Y.); 2 Research Center of 3D Printing Engineering Technology, Beijing University of Technology, Beijing 100124, China; 3 Division of Food Systems and Bioengineering, University of Missouri, Columbia, MO 65211, USA; jing.zhou@mail.missouri.edu; 4 Division of Plant Sciences, University of Missouri, Columbia, MO 65211, USA; yehe@missouri.edu (H.Y.); nguyenhenry@missouri.edu (H.T.N.)

**Keywords:** 3D imaging system, soybean, flood stress, vegetative growth

## Abstract

Flood has an important effect on plant growth by affecting their physiologic and biochemical properties. Soybean is one of the main cultivated crops in the world and the United States is one of the largest soybean producers. However, soybean plant is sensitive to flood stress that may cause slow growth, low yield, small crop production and result in significant economic loss. Therefore, it is critical to develop soybean cultivars that are tolerant to flood. One of the current bottlenecks in developing new crop cultivars is slow and inaccurate plant phenotyping that limits the genetic gain. This study aimed to develop a low-cost 3D imaging system to quantify the variation in the growth and biomass of soybean due to flood at its early growth stages. Two cultivars of soybeans, i.e. flood tolerant and flood sensitive, were planted in plant pots in a controlled greenhouse. A low-cost 3D imaging system was developed to take measurements of plant architecture including plant height, plant canopy width, petiole length, and petiole angle. It was found that the measurement error of the 3D imaging system was 5.8% in length and 5.0% in angle, which was sufficiently accurate and useful in plant phenotyping. Collected data were used to monitor the development of soybean after flood treatment. Dry biomass of soybean plant was measured at the end of the vegetative stage (two months after emergence). Results show that four groups had a significant difference in plant height, plant canopy width, petiole length, and petiole angle. Flood stress at early stages of soybean accelerated the growth of the flood-resistant plants in height and the petiole angle, however, restrained the development in plant canopy width and the petiole length of flood-sensitive plants. The dry biomass of flood-sensitive plants was near two to three times lower than that of resistant plants at the end of the vegetative stage. The results indicate that the developed low-cost 3D imaging system has the potential for accurate measurements in plant architecture and dry biomass that may be used to improve the accuracy of plant phenotyping.

## 1. Introduction

Soybean [1] is the second largest crop grown in the United States with 36.4 million ha planted, yielding US$ 40.0 billion in 2017 (USDA-ERS, 2018). However, soybean cultivars are generally sensitive to flood stress [2], which affects the physiologic and biochemical processes of soybeans, resulting in the reduction of the accumulation of dry matter (biomass) and seed yield. It was reported that approximately 16% of soybean production has been reduced due to flood stress worldwide [3]. There is a pressing need to develop high-yielding soybean cultivars with flood-tolerant trait at the current situation of rapid climate change, shortage of arable land and the increase of food demand [2]. 

Many researchers have studied the morphological responses of crops under flood stress. The formation of adventitious roots is highlighted as a common response of flood-tolerant species [4]. Flood tolerant crop cultivars often grow taller under flood conditions than they are in controlled environments, however, the biomass accumulation is lower than those under control [5,6]. After suffering flood stress, the increase in the petiole angle is the first visible symptom of the tolerant cultivars, followed by the increase in petiole length [5] in order to maximize the leaf area above the water [7]. Yield reduction was reported in soybean breeding or genetic studies when soybean suffers flood stress at different growth stages, for example, a 17–40% reduction was due to flood during vegetative stages and a 40–57% reduction during reproductive stages [8]. 

In modern soybean breeding programs, the stress tolerant cultivars are selected by planting diverse germplasms in stress environments using the criterion of the minimum yield reduction [9,10], which is extremely time-consuming (five to eight years for both cases). C. Wu et al. [10] proposed two morphological traits, namely foliar damage score and plant survival rate as the indicators of flood tolerance and they were highly correlated with grain yield (*r* = 0.95 and 0.95, respectively, *p* < 0.0001). However, the visual rating is labor-intensive and subjective due to human vision. 

Currently, the state of art plant-phenotyping technologies allows measuring crop morphological traits in an efficient and effective manner. An et al. [11] developed a high-throughput imaging system that is able to measure leaf length and area of rosette plants in two-dimensional (2D) nadir-view images. However, this platform is not able to measure plant height that is a critical trait in differentiating flood-tolerant cultivars. Jianfeng Zhou et al. [12] developed a low-cost phenotyping platform to estimate plant height of soybeans under salt-stress, and the results showed a good agreement (*R^2^* = 92% and root mean square error = 9.4 mm) between the manual and automated method. However, it is difficult to measure the leaf (petiole) angle of single soybean plant because the overlapping effects were observed between adjacent plants at the 14th day after emergence and stems were hard to be generated in three-dimensional (3D) point cloud data [13] when soybean were at early vegetation stages. Therefore, a low-cost 3D imaging system with high precision (millimeter) is necessary to measure multiple morphological traits of soybean plant under flood stress. 

To quantify crop geometric traits, O’Neal et al. [14] developed a 3D desktop scanner with open-source software to estimate leaf height and width, however, only can these two traits be measured after manually separating all the leaves from plant stems. Paulus et al. [15] developed a 3D laser scanning system to measure leaf area, stem height and plant volume of a barley plant in sub-millimeter precision. Multiple morphological plant parameters were simultaneously derived from one scanning process, and measurements were found highly correlated (*R^2^* = 0.85 − 0.97) to manually measurements, however, the laser device is not cost-efficient compared to visible imaging systems. Lou et al. [16] developed an accurate Multi-View stereo 3D reconstruction system, but it needs an expensive Canon digital camera (Canon 600D) and the reconstruction time of their method was 10–30 min depending on the number of images. Liu, Acosta-Gamboa, Huang, & Lorence [17] tested an image-based 3D plant reconstruction system for single plants. Images of plants from different views were taken using a digital camera and a turntable platform that was rotated manually. The 3D model of a single plant was successfully built using the structure from motion (SfM) method with a set of 1000 images, which is a challenge for manual image acquisition. Xiong et al. [18] established a high-throughput stereo-imaging system for the 3D reconstruction of the canopy structure of rape seedlings. The automatic measurements of leaf area and plant height agreed with the manual measurements (*R^2^* = 98.4% and 84.5% with a mean absolute percentage error of 3.7% and 6.2%, respectively). However, the binocular stereo is usually used in reconstructing canopy structures, and it will be hard to recover soybean stems where most of them are covered by canopy leaves from the top view.

The overall goal of this study was to develop a low-cost 3D imaging system to measure morphological traits of interest for quantification of the plant variations in soybean due to flood stress at early growth stages. The specific objectives were (1) to develop a low-cost imaging system for the 3D model reconstruction of soybean plants; (2) to evaluate the accuracy of the developed 3D imaging system in measuring the plant height, plant canopy width, petiole length and petiole angle of soybean plants; and (3) to evaluate the potential in estimating biomass using the extracted image features. 

## 2. Material and Methods

### 2.1. Preparation of Plants

A flood experiment was conducted at a greenhouse of the University of Missouri, Columbia, MO, USA, from May through July in 2018. Two types of soybean cultivars with known flood tolerance ability, i.e. NIL211 as the flood resistant and NIL147 as flood sensitive cultivar [19], were planted as test materials. Soybean seeds were sown in plant pots filled with pro-mix soil (PRO-MIX All Purpose Mix, Lowes, Columbia, MO, USA). After emergence, 24 plants (seedlings) with similar height and health status based on visual observation were selected from each of the two cultivars, and transplanted to 1-gal plastic plant pots (diameter = 17 cm and height = 16 cm). Plants of each cultivar were divided into two groups with 12 plants in each group, i.e. treated group (Group I and Group III for resistant and sensitive cultivars, respectively) and control group (Group II and Group IV for resistant and sensitive cultivars, respectively). At vegetative stage (V1), pots with soybean plants of Group I and Group III were put into a 3-gal plastic pots (diameter = 25 cm and height = 28 cm) and filled with water till all soil was flooded to generate submergence stress. The submergence treatment corresponds to the full saturation of the soil pores with water, and with a very thin—or even without—a layer of water above the soil surface, and only the root system of plant is under the anaerobic conditions imposed by the lack of oxygen, while the shoot is under atmospheric normal conditions [20]. The submergence treatment was conducted continuously for 10 days. Meanwhile, plants in Group II and IV were maintained under appropriate soil moisture conditions (watering three times per week) based on the daily management protocol in the greenhouse during the growing season. 

### 2.2. Development of a Low-Cost 3D Imaging System

**Figure 1:**
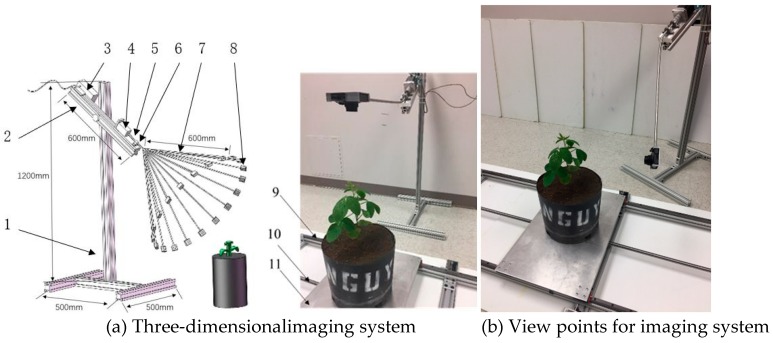
The schematic illustration of the developed three-dimensional (3D) imaging system (**a**). The components are 1-Support frame, 2-Adjustable beam, 3-Microcontroller, 4-Stepper motor, 5-Shaft coupling, 6-Bearing, 7-Camera arm at different positions, 8-Camera holder, 9-Slide rail, 10-Ball screw and 11-Transport plate. Two images on the right show the two different views of the camera.

**Figure 2:**
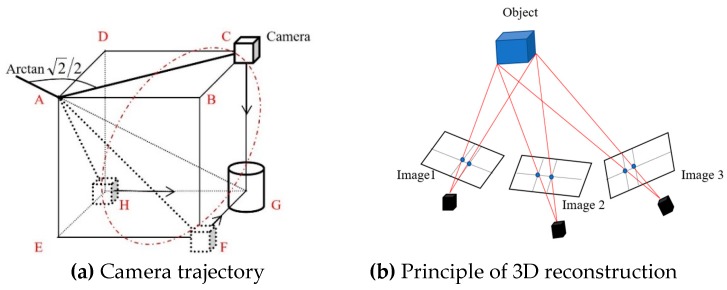
The basic principle of 3D model construction.

A 3D imaging acquisition system was developed to collect images of plants from different viewpoints to reconstruct 3D models of plant. The architecture of the imaging system is shown in Figure 1. The imaging system consisted of aluminum extrusion (50 mm × 50 mm) as the vertical support to support an adjustable aluminum beam that was used to mount a stepper motor (23HS30, www.omc-stepperonline.com), a motor driver, a micro-controller (Arduino Uno), and a bearing pair. The angle between the beam and the support is adjustable, and so as the height from the ground. A camera arm made of an aluminum stick in a diameter of 5 mm was connected to the stepper motor through the bearing pair and a shaft coupling. The arm was bent using a bending machine to a predefined angle of 145° to the center line of the motor shaft. The camera arm allowed the camera moving at a defined trajectory of a circle in a plane determined by three vertexes of C, F and H of a cube, as shown in Figure 2a. A digital camera (360HS, Canon U.S.A., Melville, NY) was mounted at the end of the camera arm using a camera house that fixed the viewpoint of the camera to the center of rotation trajectory. A control system consisted of a microcontroller (Arduino Uno R3, Sparkfun Electronics, Boulder, CO, USA) and a stepper motor driver (TB6600, SMAKN, www.dfrobot.com) was used to control the camera to move and take the images of plants from different viewpoints. The materials used for developing the imaging system were from local hardware store or low-cost off-the-shelf products. The total cost of the imaging platform, including the digital camera, aluminum stand and support, the stepper motor and control, was less than $400, the detail can be found in Supplementary Materials.

The camera was mounted at the end of the camera arm that was driven by the stepper motor to rotate in the determined plane. The camera trajectory of the 3D imaging system is shown in Figure 2a, where the dash circle through vertex points of C, F and H determined the camera motion plane. When an object (a soybean plant in this case) was placed at the corner (point G in Figure 2a) that was diagonally opposed to the corner of the bearing (point A in Figure 2a), the camera was able to take images of the object facing to the object (see the arrows of camera) with the same distance to point G. This mechanism ensured that the target object (plant) was imaged from different viewpoints and acquire uniform data points from each side of the object. The dimensions (side length, *L*_CG_) of the cube determined the imaging distances from the camera to plants, and it was found that a minimal length of 40 cm was required to cover the whole plants. The dimensions of the cube *L*_CG_ was determined by the motion trajectory of the camera, which was a function of the length of the camera arm *L*_AC_ (diagonal length, Figure 2a) shown in Equation (1):

(1) $$L_{AC}=\sqrt{2}L_{CG}$$

In this study, we selected the length of the camera arm as *L*_AC_ = 60.0 cm, resulting in the *L*_CG_ = 42.4 cm. Meanwhile, the angle of the camera arm was determined using Equation (2):

(2) $$\theta =∠\mathrm{CAG}=\tan^{-1}\left(\frac{L_{GC}}{L_{CA}}\right)=\tan^{-1}\left(\frac{\sqrt{2}}{2}\right)=145{}^{\circ}$$

**Figure 3:**
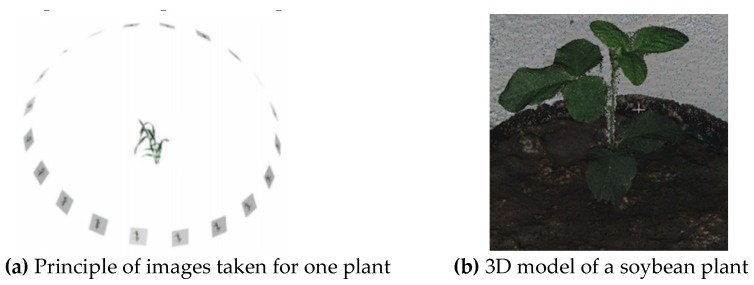
Illustration of 3D model reconstruction of a soybean plant.

The 3D models of soybean plants were reconstructed based on the method of structure from motion (SfM) that is a low-cost photogrammetric method for 3D reconstruction using a series of overlapping images. It applies a highly redundant, iterative bundle adjustment procedure, based on a database of features automatically extracted from the set of multiple overlapping images to resolve the target’s structure [21]. The arrangement of images taken from one plant as shown in Figure 3a made by Fang et al. [22]. The number of images was determined by the minimum image overlap to achieve the desired accuracy of 3D dense point and the frame rate of the snapshot of the camera. It was found that 20 images were sufficient to meet the requirement of minimal image overlap for a high-quantity 3D model in this study, which was the same as that used by Fang et al. [22]. Collected images were processed using the software Agisoft PhotoScan Pro (v1.3.4, St. Petersburg, Russia) installed on a workstation (Dell OPTIPLEX 780, CPU i7-2600, Memory 16G, solid state hard drive) to develop dense point cloud and 3D model of each plant. Figure 3b shows an example of the developed plant 3D models using the Agisoft software. In this study, it took around 20 s for completing the computing process of the 20 images and developing the 3D model of each plant. The camera was controlled to rotate at the speed of 18° per second on the defined plane (CFH in Figure 2a) to take images of a plant. The developed 3D imaging system was used to collect data for all 48 plants five times during the vegetative stages, i.e. Day 1 to Day 5 on April 2, April 4, April 6, April 8 and April 10, 2018, to acquire the temporal development information of plant under submergence stress. 

### 2.3. Extraction of Image Features 

**Figure 4:**
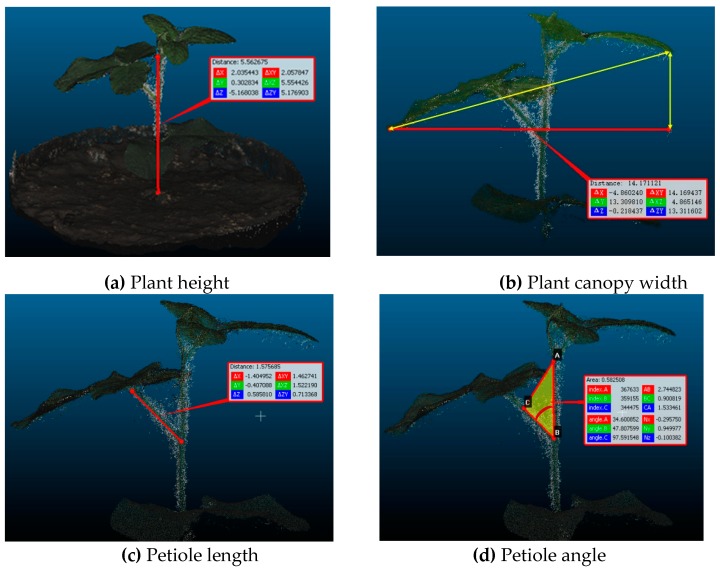
Illustration of procedure in image feature extraction using CloudCompare software.

The change in the morphological parameters of plants under different treatments was quantified using the image features of the plant height, plant canopy width, petiole length, and petiole angle, which were extracted from the 3D models of each plant on different days. The dense point cloud data were exported from Agisoft to an open source software CloudCompare (v2.9, www.cloudcompare.org) for further processing, including calibrating the dimension, removing the background (soil and plant pots) and taking measurements. The geometric calibration was conducted by comparing the measurements of the diameter of the plant pots using both the imaging system and a tape measure. The measurement of the four traits was conducted in CloudCompare manually because of the complicity of automated processing. In this study, we focused on system development and validation of usefulness of the developed system in plant phenotyping. The system will be optimized and automated processing algorithms will be developed in the future study. The measurement procedure is shown in Figure 4. Plant height was measured from the bottom of the shoot (the lowest points of the shoot) to the top of the main stem (the highest point) at the front view (Figure 4a). The plant canopy width was measured as the maximum plant canopy width from the projection of the front view (Figure 4b). The petiole length was measured as the length of the longest petiole at the front view (Figure 4c). And the petiole angle was measured as the angle between a petiole and stem (Figure 4d). Meanwhile, plant height and petiole angle were measured using a tape measure and a protractor in the 48 plants manually on April 2, 2018, which were used as ground truth to calculate the measurement accuracy in both length and angle using the 3D imaging system.

### 2.4. Soybean Biomass

To evaluate the potential in estimating biomass using the extracted image traits, soybean biomass was manually measured at the end of the vegetative stage V5 on May 18, 2018. All plant shoots were cut from the soil and were dried in an oven at the temperature of 60 °C for 120 h until completely dry. The biomass of each plant was measured using a digital weight scale with a precision of 1.0 mg. 

### 2.5. Data Analysis

All data were analyzed using the statistical toolbox of Minitab 18.0 (Minitab, LLC. State College, PA, USA), including linear regression analysis, analysis of variance (ANOVA). The measurement accuracy of plant height and petiole angle from the imaging system was calculated by comparing the data collected manually, and a linear regression analysis was conducted. 

In this study, imaging data were collected on five days during stage V1, i.e., 2nd, 4th, 6th, 8th and 10th of April, 2018, which were divided into four growth periods, i.e., 2–4, 4–6, 6–8 and 8–10. To quantify the amount of plant growth in the four growth periods, change rate (CR) in each growth period of each plant was calculated using Equation (3) for each image feature.

(3) $$CR=\;\frac{Data\left(i+1\right)-Data\left(i\right)}{Data\left(i\right)}$$

where *Data* are the plant height, plant canopy width, petiole length, and petiole angle on the *i* = 1, 2, 3, 4 or 5 time of data collection. Then the average change rate of the plant height, plant canopy width, petiole length, and petiole angle in four groups was calculated using all plants in each group. At the end of the vegetative stage V5, dry biomass of 48 plants was collected, and a one-way ANOVA was conducted to evaluate the significance in the difference between four groups. The average change rates of the plant height, canopy width, petiole length, petiole angle and dry biomass were used to explore the variation of growth and accumulation of biomass at the early vegetative stage (V1).

## 3. Results

### 3.1. Evaluation of Sensor Measurement

**Figure 5:**
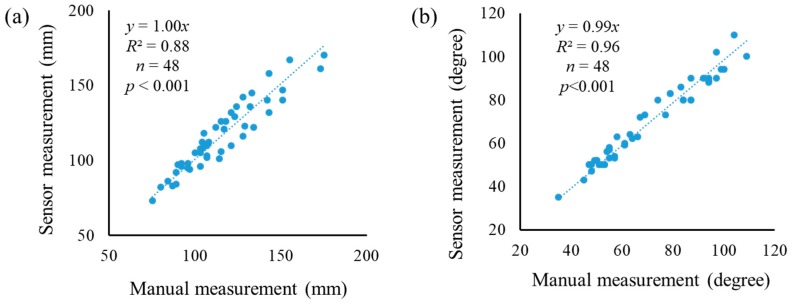
The agreement between manual measurements and image measurements at April 2. (**a**) Plant height; (**b**) petiole angle.

The agreement between the image measurements and the manual measurements was shown in Figure 5. There are strong correlations (*R*^2^ = 0.88 and 0.96) for plant height and the petiole angle at a 0.05 significance level, indicating that the 3D imaging system has potential in measuring morphological traits of interest for soybean plants at early stages.

### 3.2. Variation in Plant Development

**Figure 6:**
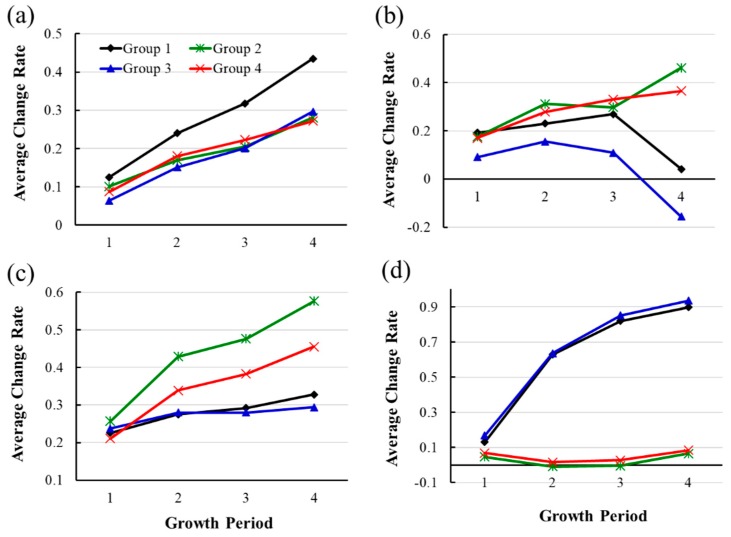
The average change rate of plants during four growth periods. (**a**)–(**d**) are the average change rates at four growth stages for the plant height, the canopy width, the petiole length and the petiole angle, respectively.

The average change rates of the four traits (i.e., plant height, plant canopy width, petiole length, and petiole angle) at four growth stages of all groups were shown in Figure 6, and the results of statistical analysis are shown in Table 1. It can be seen from Figure 6a that the plants of the flood-tolerance cultivar (Group I) under flood stress was significantly taller (*p*-value < 0.05) than that under controlled environment and the flood-sensitive cultivar under both conditions, which is consistent with the observations of Cox et al. (2003). 

The average change rates in plant height of the other three groups were similar in each growth period. On the other hand, the same flood treatment did not affect the growth of the sensitive cultivar (Group III) and no significant difference was observed in the plant height between the two cultivars in the controlled groups (Group II and IV). 

Figure 6b shows that the average change rate of the canopy width of the four groups in the four growth periods. It can be seen from the figure that the average change rates of the plant canopy width in Group I and Group III were lower than the other two control groups in most of the growth periods, especially the treated-sensitive cultivar (Group III) that was the bottom curve in all growth periods. The average change rates of the plant canopy width in Group III were significantly lower than that in the control in growth, which may be caused by the limited extension of leave span, smaller petiole length or petiole angle, indicating that the sensitive cultivar suffered more flood stress than the tolerance one. 

Figure 6c shows the impact of flood stress on the growth of the petiole length. Starting from the 2nd growth period, the average change rates in petiole length of the plants in the treated groups (Group I and III) were lower than that in the controlled groups. Statistical analysis in Table 1 shows that the means of average change rates in Group I and III were significantly lower than those of plants in Group II and IV in growth period 3 and 4. However, no significant difference was found between two cultivars in both treated and control groups.

Figure 6d illustrates the variation of average change rate in the petiole angle of plants in all groups in the four growth periods. It can be seen that the means of average change rates were clustered by cultivars, but not by treatment. The figure also shows that the petiole angle of plants in flood sensitive cultivar kept very similar due to the average change rate, which was close to zero. However, the petiole angle of plants in the flood-resistant cultivar kept increasing, which was consistent with the observations by Cox et al. [5] and Heydarian et al. [6] that the increase in the petiole angle is observed in flood-tolerant cultivars. The results also provide another quantitative measurement to distinguish the response of resistant and sensitive plants to flood stress. 

In summary, flood on soybean plants at vegetative stage V1 had a significant effect on their growth speed of plant height and petiole angle for the cultivar resistant to flood, but limited on the plant canopy width, the petiole length. Therefore, the extracted image features are able to quantify the response in soybeans in different cultivars to flood at vegetative stage V1.

### 3.3. Estimation of Biomass

The biomass of each plant and the mean of each group are listed in Table 2. The table shows that the average dry biomass of plants in the control groups (Group II and IV) was around twice or three times greater than that in the treated groups (Group I and III), respectively. A one-way ANOVA analysis shows a significant difference in biomass between treated groups and control groups (*p*-value < 0.05), however, no significant difference was found between two cultivars in the same treatment (treated or control), as shown in Table 2. The results indicate that flood stress at vegetative stage V1 had a significant impact on the accumulation of biomass, which may affect the crop development and cause yield loss.

The data of the plant height, canopy width, petiole length, and petiole angle of all 48 plants collected using the 3D imaging system on the five selected days were used to analyze the correlation between image features and the biomass. A linear regression analysis was conducted to find the regression functions of the five days. Equation (4) shows the regression function for the data of the last day.

**Figure 7:**
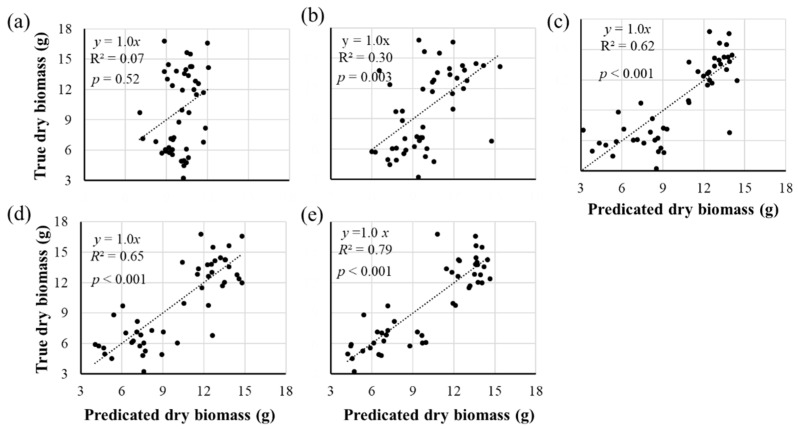
The correlations between manually measured biomass and predicated biomass using image traits collected in five days. (**a**)–(**e**) Correlations using traits extracted from the images taken on April 2, April 4, April 6, April 8 and April 10 2018, respectively.

(4) Biomass=6.68−0.002·PH+0.054·CW+0.005·BL−0.077·PA

where *PH* is the plant height, *CW* is the canopy width, *BL* is the petiole length and *PA* is the petiole angle. Figure 7 shows the correlation of the biomass measured manually and estimated with image features using Equation (4). It can be seen that there was a high correlation of the estimated values with the true values from Day 3 with the significant level at 0.001, indicating the 3D imaging system had the potential to estimate the biomass.

The statistical analysis results of the multiple linear regression analysis for Day 5 that had the highest correlation, are shown in Table 3, which indicates the model (regression) is significant, and same as the width of plant and the petiole angle. Meanwhile, the VIF shows that the collinearity of the four image features was not very high to be concerned.

## 4. Discussion

This study developed a cost-effective 3D imaging system to quantify the variation in soybean growth and biomass due to submergence stress at the early growth stage. The developed system collected plant architecture information of soybean plants, including plant height, canopy width, petiole length, and petiole angle, five times at early growth stages. Meanwhile, the biomass of each plant was measured using the oven dying method by cutting the above-ground plant shoots after the last data collection. The multiple linear regression was conducted to estimate the potential of estimate plant biomass using the developed image features. Compared with the 2D imaging system, the developed 3D imaging system is more accurate in measuring architectural information of plants and may quantify the variation of plant development due to biotic and abiotic stresses. The developed method is simple and low-cost for data acquisition compared with other 3D imaging systems. However, it also has some limitations that are needed to be further investigated, for example, inefficiency in data acquisition and image process. The developed 3D imaging system may be not suitable for a high-throughput platform [23].

The plant growth and yield of soybeans can be significantly affected by flood stress [24]. When soybean was treated with flood stress at the vegetative or the reproductive stages, grain yield and quality were reduced compared to those in control groups [25]. In soybean, cell death was detected under flood stress [26]. These findings suggest that flood causes damage to soybean at early growth stages. In this study, the effect of flood in the vegetative stage V1 showed a significant impact on the plant height and the petiole angle for the flood-resistant cultivar. However, the other architecture parameters, including the canopy width and the petiole length did not show significant differences in the growth change rate imposed by the flood stress. For the yield of soybean, it has even been suggested by Rhine et al. [27] that the influence of flood during the early vegetative period is negligible, mainly because the slower development due to flood stress at the early stages might recover during the late growth stages. Meanwhile, the flood reduced biomass by two to three times compared to the control groups in this study, which is different from the findings in Rhine et al. [27]. The potential reason might be that the soybeans were treated at different growth stages, where the treatment stage was at flowering stage for Rhine et al. [27] while plants were treated in vegetative stage V1 in this study. The results in this study are similar to the results reported by Sugimoto et al. [28], Oosterhuis et al. [25] and Scott et al. [29].

## 5. Conclusions

A cost-effective 3D imaging system was developed to take measurements of plant architecture of soybeans at the early growth stage. The developed system was evaluated in quantifying four plant traits, including plant height, plant canopy width, petiole length and petiole angle, in a flood study. The results show that flood-stress (submergence stress) during the vegetative stage V1 had significantly affected the development of soybean plant in the plant height and canopy width, which led to two times to three times reduction in the dry biomass at the end of vegetative stage V5 compared to the plants under control. The low-cost 3D imaging system was able to measure the linear length with an error of 5.78%, and angle of 4.99%, which considered accurate and useful to quantify the plant variation. On the other hand, linear regression analysis indicates that the extracted image features were able to estimate the dry biomass, especially using the data collected on the last day. This study shows that the developed 3D imaging system has the potential for the accurate measurement of plant architecture and estimation in dry biomass, and it may be useful to acquire plant traits for breeding programs. 

## Figures and Tables

**Table 1:** Results of ANOVA regarding average change rate in the plant height, canopy width, petiole length and petiole angle. The comparison was conducted among the means of different groups in each period. The different lower-case letters indicate a significant difference (*p*-value = 0.05) among the means of the average change rate in different groups in each growth period.

Average Change Rate	Period	Group I		Group II		Group III		Group IV	
		Mean	std	Mean	std	Mean	std	Mean	std
Plant height	1	0.125^a^	0.042	0.101^ab^	0.027	0.064 ^b^	0.051	0.087 ^ab^	0.039
	2	0.240 ^a^	0.047	0.170 ^b^	0.046	0.151 ^b^	0.067	0.179 ^b^	0.044
	3	0.318 ^a^	0.078	0.205 ^b^	0.049	0.201 ^b^	0.079	0.223 ^b^	0.057
	4	0.435 ^a^	0.107	0.281 ^b^	0.050	0.297 ^b^	0.079	0.272 ^b^	0.056
Canopy width	1	0.192 ^a^	0.136	0.175 ^a^	0.079	0.091 ^a^	0.128	0.170 ^a^	0.089
	2	0.230 ^ab^	0.192	0.312 ^a^	0.092	0.155 ^b^	0.130	0.279 ^a^	0.093
	3	0.269 ^ab^	0.200	0.298 ^ab^	0.249	0.109 ^b^	0.163	0.331 ^ab^	0.123
	4	0.041 ^b^	0.225	0.462 ^a^	0.128	−0.156 ^b^	0.198	0.366 ^a^	0.179
Petiole length	1	0.226 ^a^	0.099	0.257 ^a^	0.167	0.237 ^a^	0.070	0.211 ^a^	0.096
	2	0.275 ^a^	0.114	0.429 ^a^	0.205	0.280 ^a^	0.118	0.339 ^a^	0.189
	3	0.292 ^b^	0.132	0.476 ^a^	0.189	0.280 ^b^	0.118	0.383 ^ab^	0.188
	4	0.328 ^b^	0.16	0.576 ^a^	0.250	0.294 ^b^	0.112	0.455 ^ab^	0.253
Petiole angle	1	0.131 ^a^	0.133	0.046 ^a^	0.110	0.168 ^a^	0.106	0.069 ^a^	0.184
	2	0.629 ^a^	0.182	-0.008 ^b^	0.126	0.636 ^a^	0.342	0.017 ^b^	0.259
	3	0.819 ^a^	0.238	-0.004 ^b^	0.134	0.852 ^a^	0.339	0.028 ^b^	0.217
	4	0.898 ^a^	0.256	0.066 ^b^	0.200	0.936 ^a^	0.311	0.084 ^b^	0.204

**Table 2:** Biomass (g) of all the test plants. The different lower-case letters indicate a significant difference in the means of dry biomass (g) between the groups.

Plant Number	Group 1	Group 2	Group 3	Group 4
1	8.17	12.61	5.57	11.69
2	6.06	14.46	5.89	14.26
3	9.73	11.48	8.80	13.77
4	6.85	12.01	4.97	16.76
5	7.15	13.56	4.79	13.02
6	6.06	12.38	4.49	15.66
7	5.73	14.17	3.21	12.82
8	6.23	13.99	7.05	13.39
9	5.74	19.81	4.90	13.83
10	7.13	15.50	5.24	14.27
11	6.10	16.57	7.26	9.74
12	9.95	12.78	6.79	11.96
Mean	7.05 ^b^	14.11 ^a^	5.75 ^b^	13.43 ^a^

**Table 3:** The results of the statistical analysis of the multiple linear regression model using data collected on the last day.

Source	DF *	Adj SS *	Adj MS *	*F*-Value	*p*-Value	VIF *
Regression	4	591.0	147.7	29.20	0.000	--
Plant height	1	0.0	0.0	0.00	0.947	1.77
Canopy width	1	74.6	74.6	14.75	0.000	2.59
Petiole length	1	0.0	0.0	0.00	0.951	2.10
Petiole angle	1	62.1	69.2	13.68	0.001	2.22
Error	43	217.6	5.1			
Total	47	808.5				

* Abbreviations: DF: Degrees of freedom, Adj SS: Adjusted sums of squares, Adj MS: Adjusted mean squares, VIF: Variance inflation factor.

## Supplementary Materials

The following are available online at https://www.mdpi.com/1424-8220/19/12/2682/s1.
